# Beta-interferons in multiple sclerosis

**DOI:** 10.4103/0972-2327.74189

**Published:** 2010

**Authors:** Sourabh Aggarwal, Vishal Sharma, Jesna S. Mathew

**Affiliations:** University College of Medical Sciences, New Delhi, India

Sir,

We read the article “Beta-interferons in multiple sclerosis: A single center experience in India”[1] by Gupta *et al*. with great interest. We applaud the authors for conducting such a study in Indian setup. However, the high cost of therapy with interferon and even glatiramer acetate is a matter of concern in treatment of patients with multiple sclerosis in Indian setup. A study by Singhal *et al*. has concluded that mitoxantrone, which is a cheap alternative to interferon, has shown promising results in Indian setup when given as an initial therapy for the patients with multiple sclerosis[2] and could be given a try, especially when cost involved is a concern for the patients. Mitoxantrone acts due to its immunomodulatory properties by decreasing selectively the secretion of few cytokines like interferon (IFN)-γ, tumor necrosis factor-α, and IL-2.[3] Mitoxantrone has also been found to be better when used as an induction therapy followed by the use of glatiramer acetate than when glatiramer acetate was used alone in the study by Vollmer *et al*.[4] Although few adverse side effects were associated with the therapy by mitoxantrone like leucopenia, urinary tract infection, and mild hair loss.[2]

The new emerging therapies for the treatment of multiple sclerosis include alemtuzumab, natalizumab, and autologous stem cell transplantation which have also shown promising results in trials with respect to disability improvement. A study done by Cole *et al*. reported reduced disability in patients treated with alemtuzumab.[5] A multicentric study carried out by Putzki *et al*. showed significant improvement in disability score with use of natalizumab.[6] Another study using autologous hemopoietic stem cell transplantation in relapsing–remitting MS showed significant improvements in neurological disability.[7] However, all these therapies are in preliminary stage and are associated with significant high cost which is a limiting factor in treatment of patients with multiple sclerosis in India.

Nonetheless, the authors have done a commendable job in view of the patients who seriously impaired by multiple sclerosis and we appreciate their efforts.
